# Universality and superiority in preference for chromatic composition of art paintings

**DOI:** 10.1038/s41598-022-08365-z

**Published:** 2022-03-11

**Authors:** Shigeki Nakauchi, Taisei Kondo, Yuya Kinzuka, Yuma Taniyama, Hideki Tamura, Hiroshi Higashi, Kyoko Hine, Tetsuto Minami, João M. M. Linhares, Sérgio M. C. Nascimento

**Affiliations:** 1 Department of Computer Science and Engineering, Toyohashi University of Technology, Toyohashi, 441-8580 Japan; 2 Centre of Physics, Gualtar Campus, University of Minho, Braga, 4710-057 Portugal

**Keywords:** Human behaviour, Sensory processing, Social behaviour, Visual system, Colour vision

## Abstract

Color composition in paintings is a critical factor affecting observers’ aesthetic judgments. We examined observers’ preferences for the color composition of Japanese and Occidental paintings when their color gamut was rotated. In the experiment, observers were asked to select their preferred image from original and three hue-rotated images in a four-alternative forced choice paradigm. Despite observers’ being unfamiliar with the presented artwork, the original paintings (0 degrees) were preferred more frequently than the hue-rotated ones. Furthermore, the original paintings’ superiority was observed when the images were divided into small square pieces and their positions randomized (Scrambled condition), and when the images were composed of square pieces collected from different art paintings and composed as patchwork images (Patchwork condition). Therefore, the original paintings’ superiority regarding preference was quite robust, and the specific objects in the paintings associated with a particular color played only a limited role. Rather, the original paintings’ general trend in color statistics influenced hue-angle preference. Art paintings likely share common statistical regulations in color distributions, which may be the basis for the universality and superiority of the preference for original paintings.

## Introduction

A scientific approach to the aesthetic experience began with Gustave Fechner’s work on the golden section hypothesis^1^ and was followed by numerous contributions from psychological, neuroaesthetic, and theoretical studies. Since the aesthetic experience or perception of beauty is often associated with artwork^2^, the underlying brain mechanisms in the aesthetic process for artwork have been discussed extensively^3–6^. More recently, the field of neuroaesthetics has grown and expanded to involve non‑invasive brain stimulation^7^ to investigate the brain–behavior causal relationship and the computational aesthetic approach^8,9^ to bridge the gap between experimental and theoretical studies. Although studies conducted to date have generally suggested that various regions along the visual dorsal and ventral pathways are involved in the aesthetic experience, no unified view has been reached^10,11^.

Another approach to the aesthetic experience includes analysis of visual features or artwork representations, such as spatial frequency structure^12^, artistic ambiguities and conventions^13^, representation of specular reflection^14^, effects of the observers’ gaze^15^, spatial composition^16^, and efficiency of visual information coding^17^. Since color is one of the most relevant components of art paintings and may carry essentially different valence than other visual aspects like shape^18^, there have been many attempts to analyze paintings’ color to understand the brain’s aesthetic processing. For example, it was found that the color gamut of different artists’ paintings tends to exhibit the similar blue-yellow elongation found in the color gamut of typical natural environments^19,20^. The effects of color contrast, especially by a pair of complementary colors, play an important role in paintings^13^. An analysis of the chromatic composition of paintings by van Gogh was conducted to estimate his color palettes, which succeeded in detecting his usage of complementary color contours in paintings^21^.

Although attempts to analyze art paintings shed light on artistic strategies contributing to aesthetics, an analysis itself does not provide direct connections to the aesthetic experience because these studies do not include aesthetic judgment. Instead, this line of research is based on the hypothesis that art paintings that survive the selective pressures exerted by cultural institutions, collectors, and fads should contain relevant and effective visual stimuli to the nervous system^10^. The experimental approach to aesthetic judgment aims to measure observers’ responses such as like/dislike or beautiful/ugly and to investigate the causes/reasons for those responses. Not limited to paintings, there have been empirical studies on preference for complex objects such as architecture^22^ or mathematics^23^. Nevertheless, since visual artwork is composed of visual elements arranged in space, there are numerous studies on aesthetic responses to such visual elements, for example, preference for the aspect ratio of a rectangle in Fechner’s experiment^1^. It has been argued that people prefer horizontal and vertical lines rather than oblique ones^24^, curved-contour objects rather than sharp-angled ones^25^, and symmetrical shapes rather than asymmetrical ones^26^, and tend to look at the symmetrical center of images^27^. A recent computational study^28^ demonstrated that a simple linear summation of low-level visual features, such as mean hue and mean contrast, could predict observers’ subjective value ratings for art paintings.

Of these visual elements, color has accumulated the most scientific knowledge on preference^29^, because color preference is an important visual experience that influences a wide range of human behaviors. It has been argued, however, that experimental research on color preference is “chaotic”, in that inadequate measurement control has led to disparate results^29,30^. Recent studies have suggested more general hypotheses or explanations for color preference. For instance, it was argued that a universal preference for blue hues exists^30–34^. A similar tendency was reported in animals such as rhesus monkeys^35^, pigeons^36^, and zebrafish^37^, but there were more individual differences in chimpanzees^38^. Since one of the most fundamental issues of color preference is the existence of a universal tendency toward preference for a specific color^39^, the blue preference bias has attracted researchers’ attention, and there have been attempts to explain why people generally like blue more than other colors. One hypothesis is that color preference is deeply influenced by natural colors which convey biologically important signals^40^. Taking this hypothesis further, the theory of evolutionary adaptation of the visual system has proposed that color preferences are wired into the visual system as weightings on a cone opponent neural signal to improve performance on important tasks^30^.

Linked to these theories, another explanation was proposed based on ecological valence^33^, arguing that color preference arises from people’s affective responses to color-associated objects. Indeed, some claim that systematic individual differences in color preferences exist. Cultural differences in color preference have been revealed by simple pairwise comparison experiments^30^, showing that women’s preferences tend toward reddish hues whereas men’s preferences tend toward blueish hues, although these tendencies are modulated by the cultural context. Additionally, it was argued that sex differences in color preference may arise from socialization or cognitive gender development rather than inborn factors^41^. The ecological valence theory^33^ was proposed to explain and predict the individual differences in color preference. For example, this theory explains that color preferences of undergraduate students are caused by learned affective responses to university colors^42^. Other related research^43^ suggests that cultural differences in color preference can also be explained by culture-specific associations with colored objects. In contrast, some claim that there exists a universality in color preferences. Several experimental and theoretical studies have implied that color preferences are, to a considerable extent, independent of personal factors, and seem to have a firm biological basis^29^. However, neither the extent to which color preferences are universal nor the extent to which they are influenced by culture or gender is understood well.

Another issue with studies on color preference is that most only deal with a single color or a combination of few colors. Even for a combination of two colors, it is quite difficult to identify the general preference for multiple color combinations; that is, preference for color pairs cannot be predicted consistently by a preference for a single color^44^. Since art paintings generally contain numerous colors, there is a tremendous gap between preferences for a single color and a range of colors, it seems futile to approach color preference in art paintings. However, more recent studies^45–47^ have measured observers’ preferences for color composition by rotating the color gamut within the CIELAB color space. In these studies, observers were asked to adjust the hue angle of the color gamut of unfamiliar paintings to obtain the best subjective visual impression or to select the preferred one among pairs of hue-rotated versions of the same painting. These studies found that observers typically prefer a chromatic composition very close to the original rather than one that is hue-rotated, even for spatially scrambled paintings^46^. Based on those results, we suggest the following hypothesis: a universal preference for color composition in art paintings exists, and it is independent of observers’ art knowledge or experiences.

However, there are still several unsolved issues: (a) how robust is the original paintings’ superiority regarding preference for the color composition of art paintings, (b) how culturally dependent are the preference data, (c) to what extent do the spatial configuration or figurative elements of paintings influence color preference, and (d) to what extent are the statistical features or regularities of color distribution shared among art paintings. Thus, this study aims to explore these issues underlying the original-preferred judgment for art paintings by adopting a simple four-alternative forced choice (4AFC) paradigm to measure the preference for color composition of art paintings. To investigate the cultural dependency of preference for the color composition of Japanese and Occidental art paintings, observers were recruited in Japan and Portugal. Observers were asked to select their most preferred image among an original and three hue-rotated versions of the paintings (90, 180, and 270 degrees in hue angle) generated by the same procedure as the previous study^45^. Additionally, to investigate the effects of figurative elements and spatial context on preference, the spatial scrambling condition was introduced, in which paintings were divided into a set of small square pieces and scrambled in their position. This generates spatial context-free images with the same color distribution as the original painting^46^. Finally, to explore the commonality of color distribution statistics in art paintings, we measured preferences for “patchwork” images consisting of small square pieces collected from a different set of paintings. If common features in the color distribution of the art paintings exist, then the patchwork images, as the subset of a group of art paintings, should have similar color distribution features to paintings. If this is the case, and if the color distribution is the main determinant of hue-angle preference, then we can expect the hue-angle preference to show similar trends for patchwork, scrambled, and original images. Therefore, comparing hue-angle preferences under these conditions should provide important information on the effects of spatial configuration and color distribution in art paintings on hue-angle preference.

## Materials and methods

### Paintings

Twenty paintings (ten Occidental and ten Japanese) which were digitized at the Centro de Arte Moderna da Fundação Calouste Gulbenkian, Lisboa, Portugal; the Museu Nogueira da Silva, Braga, Portugal; the color laboratory of the University of Minho, Braga, Portugal; and Toyohashi City Museum of Art and History, Toyohashi, Japan were used. They were measured using a hyperspectral camera to estimate spectral reflectance and reproduce RGB images illuminated by daylight with the correlated color temperature (CCT) of 6500 K (D65) as the visual stimuli displayed on calibrated monitor screens (for more details, see the previous studies^45,48^). The Occidental paintings were oil paintings dating from the Renaissance to the modern period (fourteenth–twentieth centuries). Japanese paintings were created during the late Edo period up to the modern period (eighteenth–twentieth centuries), all of which used traditional Japanese paper (*washi*) and pigments derived from natural ingredients such as minerals, shells, or corals (*iwa-enogu*). In addition, image data from 20 paintings (ten figurative and ten abstract) were collected from three art painting galleries on the Internet (*The Metropolitan Museum of Art*^49^, *Web Gallery of Art*^50^, *WikiArt*^51^*,* and *Wikimedia Commons*^52^) to use as visual stimuli in the experiments. For paintings measured in museums, we selected the paintings that could be successfully captured by a hyperspectral camera, because some paintings in museums, especially Japanese paintings, contain materials with specular reflections, such as gold leaf, which cause dynamic range problems. Additional paintings in the Internet gallery were collected using a random principle, from figurative and abstract genres; paintings with only a few colors or using only achromatic colors were eliminated manually from the collected samples.

**Figure 1:**
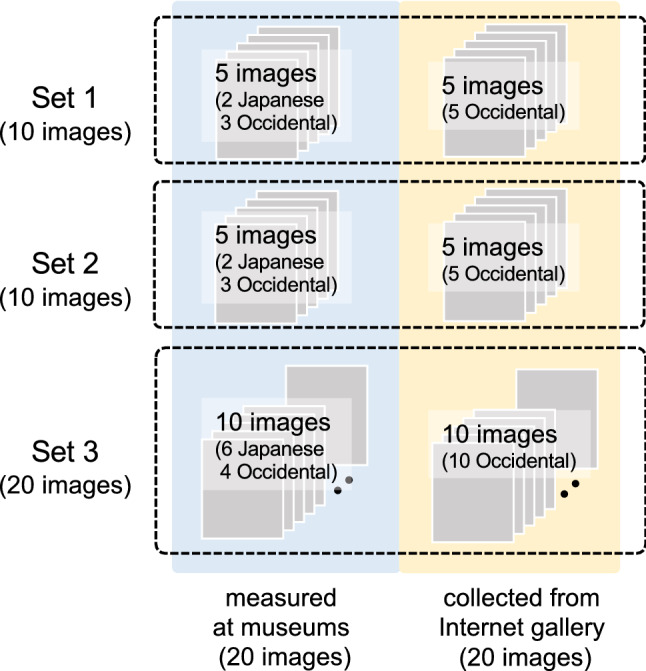
Paintings used in the experiments. Ten Japanese and ten Occidental paintings were digitized at museums or laboratories in Japan and Portugal using a hyperspectral imaging technique. In addition to these, 20 more paintings were added from art galleries on the Internet. The total of 40 images of paintings were divided into three sets, in which Sets 1 and 2 comprised ten images each, and Set 3 comprised 20 images. These image sets were used in different experimental conditions. Specific information on the paintings is given in Table 1.

As shown in Fig. 1, the 40 images of paintings listed in Table 1 were divided into three image sets: two sets (Set 1 and Set 2) with ten images each, including five paintings from museums (two Japanese and three Occidental paintings) and five paintings (all Occidental paintings) from art painting galleries on the Internet; and the third (Set 3) with ten paintings from museums (six Japanese and four Occidental paintings) and ten paintings (all Occidental paintings) from art painting galleries on the Internet. Paintings in these sets were selected using a random principle, and used in different experimental conditions (C0, C1, C2, and C3) explained below.

**Table 1:** List of paintings used in the experiment.

Set	Source	Title	Artist	Year
Set 1	Museum/Lab	Yuri	Chusaku Oyama	Late XX century
		Okao no saku	Munakata Shiko	Late XX century
		Unknown	Unknown	Unknown
		Brut (300 TSF) 2	Amadeo de Souza-Cardoso	1917
		Coty	Amadeo de Souza-Cardoso	1917
	Internet	The Adoration of the Magi	Jacopo Bassano	1569
		Beach Scene, Trouville	Eugène Boudin	1863
		Mechanical Elements	Fernand Léger	1920
		Guitar and Clarinet on a Mantelpiece	Pablo Picasso	1915
		Galisteo Creek	Susan Rothenberg	1992
Set 2	Museum/Lab	Hana	Masayoshi Nakamura	Late XX century
		Kibayo	Kazuo Omori	Late XX century
		Título desconhecido (O jockey)	Amadeo de Souza-Cardoso	1913
		Unknown	unknown	Unknown
		Unknown	Wan Kteben	Renaissance époque
	Internet	Grotto in the Gulf of Naples	Karl Blechen	1829
		Girl in a Pink Dress	Jean-Frédéric Bazille	1864
		Renaissance Interior with Banqueters	Batholomeus van Bassen	1618–1620
		No. 13 (White, Red on Yellow)	Mark Rothko	1958
		Objects on a Table	Patrick Henry Bruce	1920–21
Set 3	Museum/Lab	Fuji	Togyu Okumura	Late XX century
		Hakouomotehama	Shigeo Nagai	Late XX century
		Kanbotan	Toshio Matsuo	Late XX century
		Karasaki no yau	Hiroshige Utagawa	1832
		Sankomidori	Kaii Higashiyama	Late XX century
		Asayake Fuji	Shutaro Muramatsu	Late XX century
		Painting	Amadeo de Souza-Cardoso	1917
		Ar livre—nú (open air—naked)	Amadeo de Souza-Cardoso	1914
		unkonwn	Carlos Ramos	XIX century
		Entrada	Amadeo de Souza-Cardoso	1917
	Internet	Yosemite IV	Natvar Bhavsar	1980
		Beach Scene, Trouville	Eugene Boudin	1863
		White Rectangles, Number 3	Irene Rice Pereira	1939
		The Death of Harmonia	Jean-Baptiste Marie Pierre	1740–1741
		Renaissance Interior with Banqueters	Batholomeus van Bassen	1618–1620
		Untitled, 2013	Golnaz Fathi	2013
		Interior of a Gothic Cathedral	Batholomeus van Bassen	1614
		Redgreen and Violet-Yellow Rhythms	Paul Klee	1920
		The Annunciation to the Shepherds	Jacopo Bassano	1555–1560
		Portrait of a German Officer	Marsden Hartley	1914

### Participants

In total, 45 Japanese individuals (37 men and 8 women, aged 21–65 years, mean 26.6 years, SD 10.5 years) and 45 Portuguese individuals (13 men and 32 women, aged 17–25 years, mean 19.8 years, SD 1.8 years) completed the main experiments in either Japan or Portugal under exactly the same conditions. To clarify the stimulus dependency of preference data in the main experiment, a replication experiment was conducted with identical procedures and different visual stimuli. The participants in the replication experiment were 44 newly recruited Japanese observers (26 men and 18 women, aged 21–68 years, mean 34.8 years, SD 14.9 years old) and 44 Portuguese observers (12 men and 32 women, aged 17–25 years, mean 19.8 years, SD 1.8 years) who had also participated the main experiment. All observers were unaware of the purpose of the experiments. They had no formal artistic education and only basic knowledge about the art paintings. After they finished the experiments, observers were asked verbally about their knowledge of the paintings used as stimuli, and none of them had previous knowledge about the paintings. Undergraduate and graduate university students in Japan and Portugal were recruited to participate. All participants had normal or corrected-to-normal visual acuity and normal color vision as tested with Ishihara plates. All experimental procedures were completed according to the ethical principles outlined in the Declaration of Helsinki and approved by the Committee for Human Research at the Toyohashi University of Technology. The experiment was strictly conducted following the approved guidelines of the committee. Written informed consent was obtained from the participants after procedural details were explained.

### Experimental procedures

#### Stimuli

**Figure 2:**
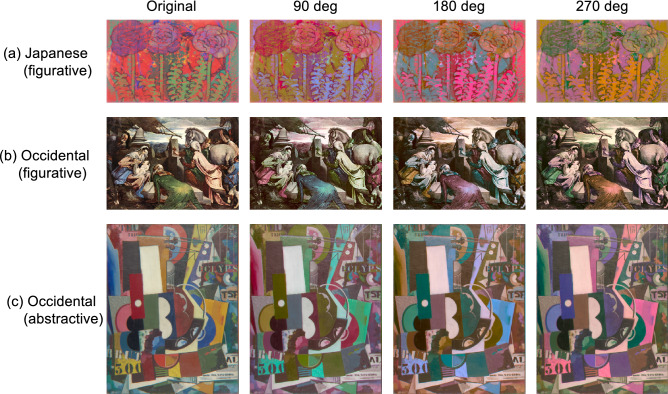
Typical examples of original and hue-rotated images used in the experiments. (**a**) Japanese painting on Japanese paper (*washi*) by *Masayoshi Nakamura* (1924–1977), late twentieth century; (**b**) oil painting on canvas by *Jacopo Bassano* (ca. 1510–1592) in 1569. (**c**) oil painting on canvas by *Amadeo de Souza-Cardoso* (1887–1918) in 1917.

The stimuli used in the experiment consisted of four types of images of the paintings, including originals, selected from Set 1 in the main experiment, and from Set 2 in the replication experiment. The standard RGB (sRGB) coordinates of each pixel of the original images were computed to display the images on calibrated monitor screens and were transformed to the corresponding color coordinates (L*, a*, b*) in the CIELAB color space. The average values of L*, a*, and b* of each original image ranged from 20.7 to 63.9, mean = 47.9 for L*; from − 15.9 to 31.9, mean = 5.21 for a*; and from − 13.9 to 46.6, mean = 12.2 for b*, respectively. The hue-rotated images were obtained by rotating the color gamut of the originals, a set of points for each pixel coordinate in the CIELAB color space, around an axis parallel to the L* axis passing through the mean chromaticity point on the a*–b* plane. The original corresponded to 0 degrees and the other three hue-rotated images were obtained by rotating the color gamut by 90, 180, and 270 degrees counterclockwise on the a*–b* plane. Figure 2 presents typical examples of the hue-rotation effect on color appearance in both Occidental and Japanese paintings. When the hue of the original images displayed on the monitor screen were rotated, some pixel colors were outside the gamut of the monitor. Colors out of the gamut were projected onto the closest displayable colors in the CIELAB color space and chromatic errors occurred for these pixels. The largest chromatic error among the hue-rotated images used in the experiments was ${\Delta E}_{ab}=3.8$, which is near the threshold for complex images^45,53^; thus, the effect of the gamut compression caused by the hue rotation was fairly negligible.

#### Experimental conditions

**Figure 3:**
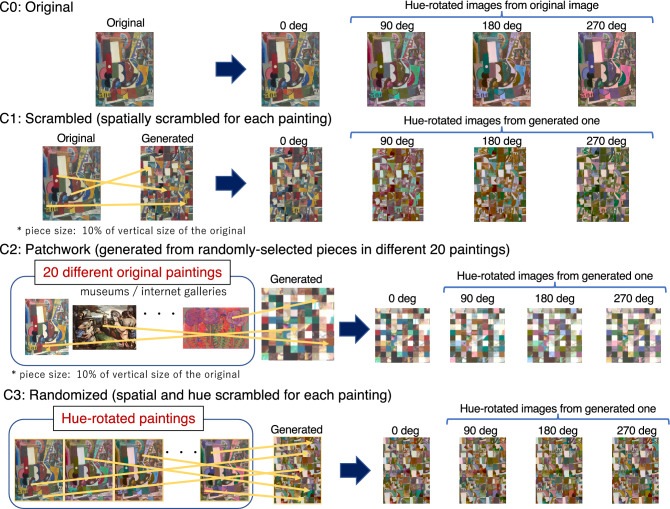
Image scrambling condition. C0 (Original), original and hue-rotated images are preserved their original spatial form. C1 (Scrambled), each image is divided into a set of small square pieces with a side length of 73 pixels (10% of the height of the image), and the position of the piece is randomized. C2 (Patchwork), each image is divided into a set of small pieces in the same way as C1 and each stimulus is created with 100 randomly selected pieces from 20 different paintings measured at museums or from the galleries on the Internet in Sets 1, 2 and 3. C3 (Randomized), each stimulus is generated from the pieces selected from the hue-rotated images with a random degree from the original image.

Some paintings used in the experiment contained familiar objects associated with specific memory colors, such as a blueish sky^54^. To demonstrate whether hue-angle preference depended on the overall color composition of the paintings or specific objects’ colors in the paintings, the spatial scramble was introduced to manipulate the spatial composition and visibility of the figurative elements of the paintings, as shown in Fig. 3. In C0 (Original condition), originals (selected from Set 1 in the main experiment and from Set 2 in the replication experiment) and hue-rotated images were preserved in their original spatial form. In C1 (Scrambled condition), each original image was divided into a set of small square pieces with a side length of 73 pixels (10% of the height of the image), and the position was randomized. The generated images were the same size as the originals and were used to create the hue-rotated images. In C2 (Patchwork condition), each image was divided into a set of small pieces in the same manner as for C1, and each stimulus was generated by composing 100 randomly selected pieces (10 × 10 pieces) from the groups of 20 different paintings measured at museums or of 20 different paintings from the galleries on the Internet, in a mixture of all three sets (Sets 1, 2, and 3). In C3 (Randomized condition), each stimulus was generated from pieces of different images similar to that in C2; however, each piece was selected from the hue-rotated images with a random degree from the original. In each condition, ten sets of four types of images, including original or generated images, were used as stimuli. In C2, five images each were generated from images measured at museums and from art painting galleries on the Internet.

In C1, C2, and C3, the original paintings were divided into small pieces (73 × 73 pixels) to make it difficult to identify any specific objects from one piece. In C1, the generated images had color compositions identical to the originals. In C2, the population of the generated images had the same color statistics as the population of the 20 original paintings. In C3, the generated images were randomized spatially and chromatically from the originals. Therefore, by comparing the results of C0 with C1, C2, and C3, it was possible to determine whether spatial compositions, figurative elements, or color composition influenced the hue-angle preference, or if it were influenced by the general tendencies of the original paintings’ color statistics.

#### Apparatus and task

The original (0 degree) and three hue-rotated images (90, 180, and 270 degrees) were displayed on a calibrated monitor in two columns and two rows, 1.7 cm apart. The viewing distance was 50.0 cm, the height of the images on the screen was fixed to 6.0 cm (730 pixels), and the width varied from 3.9 cm (478 pixels) to 11.2 cm (1365 pixels) depending on the original image size. The stimuli were displayed on a 12.4-inch screen with a resolution of 2736 × 1824 pixels (Surface Pro4, Microsoft). Observers were instructed to select their most preferred image among the four by touching the screen (4AFC). Each stimulus condition (shown in Fig. 3) included 10 image sets with original and three hue-rotated images and each stimulus set was presented only once, resulting in a total of 40 trials. The order of stimulus presentation was random. No information was provided to observers about the original paintings and hue rotation and there was no time limit to respond in each trial.

### Data analysis

The average rates of selection for each image category (original and 90, 180, and 270 degrees hue-rotated) were analyzed with a multinomial test separately for Japanese and Portuguese observers and each condition. Selection rates for the original images (0 degrees) as an index of the original paintings’ superiority were analyzed by two-way repeated-measures analysis of variance (ANOVA) for the stimulus conditions (C0, C1, C2, and C3) and observer nationality (Japanese and Portuguese) as factors. The level of statistical significance was set at *p* < 0.05 for all analyses. Within-subjects effects were corrected using the Greenhouse–Geisser correction. Pairwise comparisons for the main effects were corrected for multiple comparisons using the Bonferroni correction. Effect sizes (*η*^2^ and ${\omega }^{2}$) were determined for the ANOVA. All the statistical analyses were performed using the statistical software JASP^55^.

## Results

**Figure 4:**
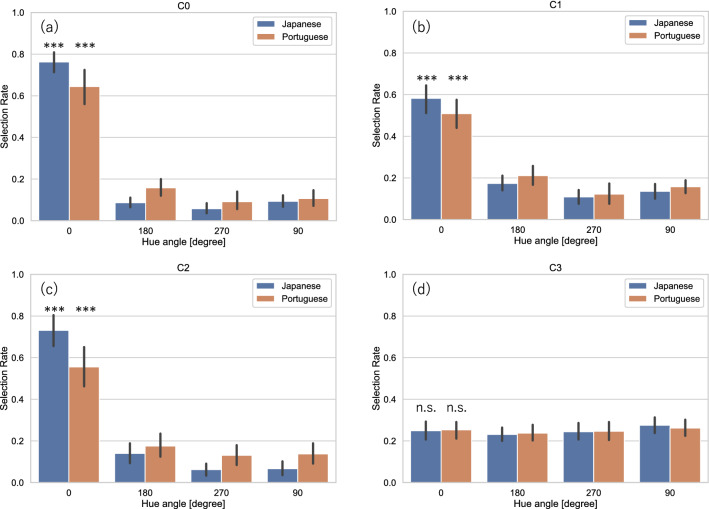
Average selection rates for original (0 degrees) and 90, 180, and 270 degrees hue-rotated images for Japanese and Portuguese observers measured in the main experiment. Selection rates measured in (**a**) C0 in which images were manipulated only with hue rotation, (**b**) C1: Scrambled, (**c**) C2: Patchwork, and (**d**) C3: Randomized conditions. Bars represent 95% confidence intervals. Asterisks indicate a significant difference from chance level; ***p < 0.001.

Figure 4 shows the average selection rates measured in the main experiment for each image category, the original and three hue-rotated images, measured from Japanese and Portuguese observers in conditions C0, C1, C2, and C3. In condition C0, in which the displayed images were preserved in their original spatial forms, the average selection rate for the original images was significantly above chance levels for both Japanese and Portuguese observers (Japanese observers: ${\mathrm{\rm X}}^{2}= 630.978; p<0.001$, Portuguese observers: ${\mathrm{\rm X}}^{2}= 377.787; p<0.001$), implying that the original images were preferred more frequently than the hue-rotated images. The original paintings’ superiority with regard to preference was observed in the spatially scrambled condition (C1), as shown in Fig. 4b, and even in the patchwork condition (C2), as shown in Fig. 4c. In both C1 and C2, the average selection rates for the original images were significantly above chance levels (C1: ${\mathrm{\rm X}}^{2}= 268.667, p<0.001$ for Japanese observers; ${\mathrm{\rm X}}^{2}= 168.062, p<0.001$ for Portuguese observers; C2: ${\mathrm{\rm X}}^{2}= 562.391, p<0.001$ for Japanese observers; ${\mathrm{\rm X}}^{2}= 226.142, p<0.001$ for Portuguese observers), depicting the same tendency found in C0. However, in C3, which randomized the images both spatially and chromatically, differences between the average selection rates and chance levels were not observed for either Japanese or Portuguese observers $({\mathrm{\rm X}}^{2}= 1.876, p=0.599$ for Japanese observers; ${\mathrm{\rm X}}^{2}= 0.578, p=0.902$ for Portuguese observers).

**Figure 5:**
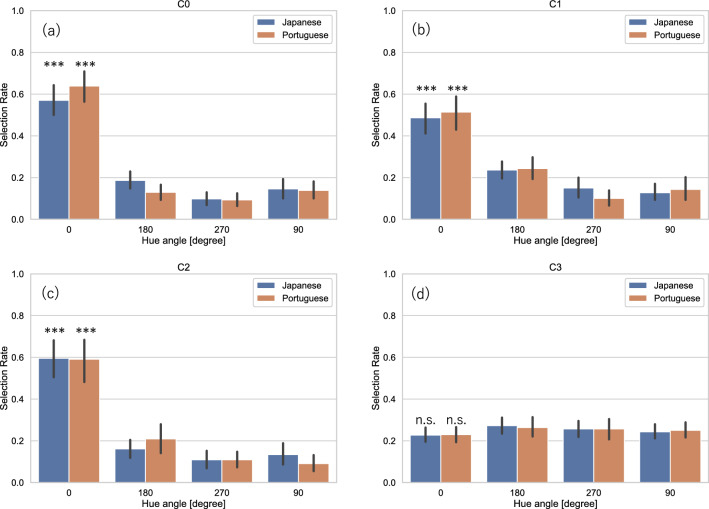
Average selection rates measured in the replication experiment. The format is identical to Fig. 4.

Figure 5 shows the average selection rates measured in the replication experiment in the same way as Fig. 4. In C0, as shown in Fig. 5a, the average selection rates for the original images were significantly above chance levels for both Japanese and Portuguese observers (Japanese observers: ${\mathrm{\rm X}}^{2}= 247.999,\mathrm{ p}<0.001$; Portuguese observers: ${\mathrm{\rm X}}^{2}= 356.473,\mathrm{ p}<0.001$), clearly replicating the results obtained in the main experiment shown in Fig. 4a. Figure 5b–d show the selection rates in C1, C2, and C3. The average selection rates for the original images were significantly above chance levels in C1 and C2 (C1: ${\mathrm{\rm X}}^{2}= 142.764,\mathrm{ p}<0.001$ for Japanese observers; ${\mathrm{\rm X}}^{2}= 182.091,\mathrm{ p}<0.001$ for Portuguese observers; C2: ${\mathrm{\rm X}}^{2}= 282.455,\mathrm{ p}<0.001$ for Japanese observers; ${\mathrm{\rm X}}^{2}= 288.982,\mathrm{ p}<0.001$ for Portuguese observers), while the differences between the average selection rates and chance levels were not observed for either Japanese or Portuguese observers in C3 $({\mathrm{\rm X}}^{2}= 1.982,\mathrm{ p}=0.576$ for Japanese observers; ${\mathrm{\rm X}}^{2}= 1.145,\mathrm{ p}=0.766$ for Portuguese observers).

**Figure 6:**
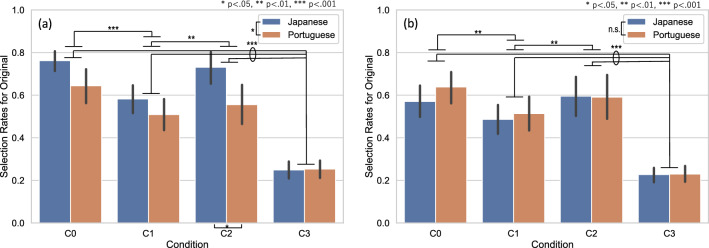
Average selection rates for the original images (0 degree) in different conditions. (**a**) The average selection rates measured in the main experiment reiterated from Fig. 4. (**b**) The average selection rates measured in the replication experiment reiterated from Fig. 5. Bars represent 95% confidence intervals. Asterisks indicate a significant difference; *p < 0.05, **p < 0.01, ***p < 0.001.

Figure 6a reiterates the selection rates for the original images measured in the four conditions in the main experiment shown in Fig. 4. ANOVA on these selection rates revealed significant main effects of condition (*F*[2.231, 196.322] = 110.213 [with the Greenhouse–Geisser correction], *p* < 0.001; ${\eta }^{2}$ = 0.354, ${\omega }^{2}$ = 0.363) and observer nationality (*F*[1, 88] = 6.500, *p* = 0.013; ${\eta }^{2}$ = 0.024, ${\omega }^{2}$ = 0.030). There was a significant interaction between condition and observer nationality (*F*[2.231, 264] = 3.944 [with the Greenhouse–Geisser correction], *p* = 0.017; ${\eta }^{2}$ = 0.013, ${\omega }^{2}$ = 0.015). A post-hoc comparison among conditions revealed that the average selection rate for the originals in C3 was significantly lower than that in other conditions: C0 (*t* = 16.731, *p* < 0.001; mean difference = 0.452, 95% CI [0.380, 0.524]); C1 (*t* = 10.894, *p* < 0.001; mean difference = 0.294, 95% CI [0.223, 0.366]); and C2 (*t* = 14.511, *p* < 0.001; mean difference 0.392, 95% CI [0.320, 0.464]). The average selection rate in C1 was lower than that in C0 (*t* = 5.837, *p* < 0.001; mean difference 0.158, 95% CI [0.086, 0.230]), and C2 (*t* = 3.618, *p* = 0.002; mean difference 0.098, 95% CI [0.026, 0.170]). There was a significant difference between the selection rates of Japanese and Portuguese observers only in condition C2 (*t* = 3.616, *p* = 0.010; mean difference 0.176, 95% CI [0.022, 0.329]).

Figure 6b reiterates the average selection rates for the original images measured in the replication experiment shown in Fig. 5. ANOVA on these selection rates revealed significant main effects of condition (*F*[1.833, 158.040] = 80.742 [with the Greenhouse–Geisser correction], *p* < 0.001; ${\eta }^{2}$ = 0.269, ${\omega }^{2}$ = 0.265), and no effects of observer nationality (*F*[1, 86] = 0.309, *p* = 0.580; ${\eta }^{2}$ = 0.002, ${\omega }^{2}$ = 0.000). There was no significant interaction between condition and observer nationality (F[1.838, 158.040] = 0.712 [with the Greenhouse–Geisser correction], *p* = 0.481, ${\eta }^{2}$ = 0.002, ${\omega }^{2}$ = 0.000). A post-hoc comparison among conditions revealed that the average selection rate for the original in C3 was significantly lower than that in C0 (*t* = 13.647, *p* < 0.001; mean difference = 0.376, 95% CI [0.303, 0.449]), C1 (*t* = 9.854, *p* < 0.001; mean difference 0.272, 95% CI [0.198, 0.245]), and C2 (*t* = 13.234, *p* < 0.001; mean difference 0.365, 95% CI [0.291, 0.438]). The average selection rate in C1 was lower than in C0 (*t* = 3.793, *p* = 0.001; mean difference 0.105, 95% CI [0.031, 0.178]), and C2 (*t* = 3.381, *p* = 0.005; mean difference 0.093, 95% CI [0.020, 0.166]).

## Discussion

The present study aimed to answer the questions underlying the original-preferred judgment for art paintings: (a) how robust is the original paintings’ superiority with regard to preference for the color composition of art paintings, (b) how culturally dependent are the preference data, (c) to what extent do the spatial configuration or figurative elements of paintings influence color preference, and (d) to what extent are the statistical features or regularities of color distribution shared among art paintings. We performed a series of 4AFC experiments using original and hue-rotated images to measure the preference for unfamiliar art paintings in Japanese and Portuguese observers by manipulating figurative elements of paintings by scrambling the images. The results showed that both Japanese and Portuguese observers preferred the originals more than the hue-rotated ones, replicating findings from previous studies^45,46^ on original paintings’ superiority regarding preference, thus confirming that the preference for the color composition of the original paintings was extremely robust. The original paintings’ superiority was observed not only in the condition that preserved the original spatial composition of paintings (Original condition) but also in the condition with spatial scrambling in which the images were divided into small square pieces and their positions were randomized (Scrambled condition), and even in the conditions that mixed the square pieces of different art paintings (Patchwork condition). These findings imply that spatial context or specific objects in paintings associated with a particular color do not play an important role in hue-angle preference. Rather, the general trend in the color statistics of the original paintings influences hue-angle preference.

We observed a significant difference in the average selection rates for the original paintings between Japanese and Portuguese observers in the main experiment, although the effect size was relatively small^56^ (${\eta }^{2}$ = 0.024, ${\omega }^{2}$ = 0.015). Although cultural dependency in preference for a single color has been reported^30^, the influence of cultural context or individual experience is complex and thus not yet clear. When we focused on the color preferences between Japanese and Portuguese observers, there was a clear difference in the preference for the CCT of illumination, showing that Japanese observers preferred a significantly higher CCT (more blueish) than Portuguese observers for illuminating art paintings^57^. Our findings indicate that original paintings’ superiority with regard to preference is common, but difference in sensitivities to the original is likely dependent on the selected paintings. We tested this idea with a different set of paintings as a replication experiment. As shown in Figs. 5 and 6, the differences in the average selection rates for the originals between nationalities were not replicated, while the original superiorities in all conditions except for the randomized condition were observed. Therefore, we could not find a clear link between cultural differences and preference level for original color compositions in our current data measured for original and hue-rotated paintings.

When comparing C1 (Scrambled condition) with C0 (Original condition), although the figurative elements were designed to disappear, the average selection rate for the originals was significantly above chance level in C1, but not as high as in C0. This suggests that the spatial context or figurative elements associated with certain colors (e.g., skin or sky) influenced the selection rate for the originals in a limited way. We examined the average selection rates measured in C0 (Original condition) for twenty images used in the experiments separately (see Supplementary Information). The top three images in terms of the average selection rates were all portraits of human faces, which could be a strong cue for original images, as shown in Supplementary Fig. S1. However, other than that, there was no dominant dependency on the type of image (figurative/non-figurative). Therefore, except for a few special cases such as human faces, the color distribution statistics, rather than spatial layout or composition of figurative elements of paintings, are considered to play a relatively important role in a preference for the originals. However, the most striking finding in the current study is that the average selection rate for the originals in C2 (Patchwork condition), where the images were composed of small, randomly selected pieces from 20 different paintings, was above chance level and more or less similar to C0. This suggests that the color distribution statistics of the original art paintings may have similar characteristics. Conversely, without this possibility, it is difficult to explain why the images composed of random pieces were preferred and became less preferred when the hue was rotated.

What are the possible statistical features or regularities in the color distribution common to the art paintings? Since a degree of preference decreased by the rotation of the color gamut, statistical features linked to the preference, if they exist, should also change at the same time. The hue rotation preserves lightness, mean chromaticity, saturation, and relative relationships between colors in the image^45^. Previous studies on preferences for a single color^30–34^ or the CCT of illumination^57,58^ have in principle manipulated the mean chromaticity of stimuli. Although the hue rotation did not change the mean chromaticity in principle, it did affect the hue angle distribution of an image. It is therefore possible that, for example, the mean of the hue angle distribution of the paintings is related to the average selection rate. However, we found no evidence that the mean of the hue distribution had any effect on the average selection rates (see Supplementary Fig. S1); thus, the preference bias in the hue domain found in previous studies for single colors^30–34^ does not explain our results here.

It seems, therefore, that the key to answering the question lies in the more general nature of the color gamut of art paintings. An analysis of the 2D shapes of the color gamut of art paintings and natural scenes found that the color gamut of art paintings had shapes elongated in the yellow-blue directions and this feature was partly shared with natural scenes^19,20^. The hue rotation directly changed the hue angle of the color gamut. One possible explanation is that artists imitate, implicitly or explicitly, the naturalistic aspect of the color gamut of natural scenes (e.g., elongating in yellow-blue direction) in their paintings, and they intuitively know that this naturalistic feature matches observers’ preferences. This was supported by a recent study^59^ which showed that images perceived as more natural were preferred. However, “naturalness” does not necessarily mean resemblance to natural scenes; hence, the question of “regularity of color distribution determining the preference/naturalness” remains open^20,59^.

It is likely plausible that the artist intuitively understands observers’ preferences if there exists a universal form regarding preference for color composition. The hue rotation does change the relationship between lightness and colors. If there exists a naturalistic link between lightness and color, and observers intuitively know this relationship, then it is plausible that observers will notice when this naturalistic relationship is violated by the hue rotation. These kinds of regulations or constraints between lightness and colors may derive from the materials used in the paintings, such as pigments and dyes^60^. A more cognitive explanation might concern the color categories; observers prefer images in which more colors can be categorized as typical colors because such images might be processed more fluently, as posited by the fluency theory^26^ and argued in a recent study^61^. Although further studies are required to clarify these unsolved issues, as this study showed, the phenomenon that the original paintings were preferred more than other hue-rotated ones was quite robust, and the original paintings’ superiority regarding preference was preserved even for the patchwork images composed of different original paintings.

Our study has three main limitations. First, according to our experimental design, obtained selection rate data reflect relative preferences among four types of images, including original images. Manipulation of hue angles with preserving lightness and mean chromaticity in the current experiment succeeded in identifying the effects of color composition on preferences and showing the original paintings’ superiority regarding preference. However, the question remains as to what extent color composition, compared to other visual features, influences preferences across different paintings. It has been reported that preferences for paintings were well-predicted by preferences for the objects depicted in the paintings^62^, although this cannot explain preferences for paintings without objects, such as abstract paintings. More recently, it has been demonstrated that a linear combination of low-level visual features (12 global and 28 local features) can predict preferences for both paintings and photography^28^. This implies that there exist universal visual features that are relevant to aesthetic judgements. Further research could explore the details of links between color features and aesthetic judgement as a basis for original paintings’ superiority.

Second, we did not find a clear relationship between cultural differences and preferences for the original color compositions. Although we found differences in the average selection rates for originals between Japanese and Portuguese observers measured in the main experiment, as shown in Fig. 6a, it could be possible that these differences were dependent on gender, as the ratio of men to women significantly differed between the Portuguese and Japanese groups $\left({\mathrm{\rm X}}^{2}= 25.920, p<0.001\right)$. However, a replication experiment, shown in Fig. 6b, demonstrated no significant differences in the average selection rates between two nationalities, even when there was a similar gender imbalance in the observer groups $\left({\mathrm{\rm X}}^{2}= 9.078, p<0.01\right)$. To explore the effects of culture and gender on preference, further studies to compare more cultures with gender-balanced observers are required. In addition to this, there was a different trend in the selection rates of Japanese and Portuguese participants in the main and replication experiments. The selection rates of the same Portuguese participants in the two experiments were almost the same. In the case of the Japanese participants, however, the selection rates of the different samples in the two experiments were more varied. This suggests that, in addition to cultural differences, there may exist remarkable individual differences, although it was not possible to clearly distinguish the effects of these factors because of the difference between the samples in the two experiments.

Third, the number of paintings used in the experiments was limited. In the present study, we used 20 paintings (Set 1 and Set 2) in conditions C0, C1, and C3, while another 20 paintings in Set 3 were used only for generating patchwork images in C2. The 40 paintings comprised 10 Japanese paintings and 10 occidentals measured in museums, and 20 occidentals collected from Internet art galleries. In the future, paintings with more varied cultural backgrounds are necessary to examine in depth the cultural dependency in color preference of paintings, and to directly test whether similarity of paintings’ color statistics to natural scenes affects the preference for chromatic composition of paintings. These experiments are being conducted as part of our upcoming research.

In conclusion, the findings of this study imply that original paintings’ superiority is quite robust, and art paintings likely share common statistical regulations in color distribution, which should be an important argument for further clarifying the underlying mechanisms for color preference of art paintings. Determining whether universal color features are a basis for preference judgements will shed light on the developmental origins of the human color vision system and its biological value.

## Supplementary Information

Supplementary Information.

## Data Availability

All datasets generated during this study are included in this article or the analyzed data are fully available from the corresponding author on reasonable request.
