# Supplementary material for: Universality and superiority in preference for chromatic composition of art paintings
Source: Sci Rep. 2022 Mar 11;12:4294. doi: 10.1038/s41598-022-08365-z (PMC8917196; doi:10.1038/s41598-022-08365-z)
Supplement: Supplementary file 1 — Supplementary Information. [file 41598_2022_8365_MOESM1_ESM.pdf]

Supplementary information

Universality and superiority in preference for chromatic composition of art paintings

Shigeki Nakauchi[1*], Taisei Kondo[1], Yuya Kinzuka[1], Yuma Taniyama[1], Hideki Tamura[1], Hiroshi Higashi[1], Kyoko Hine[1], Tetsuto Minami[1], João M.M. Linhares[2], Sérgio M.C. Nascimento[2]

[1]Department of Computer Science and Engineering, Toyohashi University of Technology, Toyohashi 441-8580, Japan

[2]Centre of Physics, Gualtar Campus, University of Minho, Braga 4710-057, Portugal

***Corresponding author**

Email: nakauchi@tut.jp

Results' dependency on image types and mean of hue distribution

We first checked the dependency of the average selection rates on painting type. We analyzed the selection rates for each image measured in the condition C0 (Original condition) of the main experiment (N=45) for Set 1, and in the replication experiment (N=44) for Set 2. The top three images in terms of the average selection rates were all portraits with human faces, which may be a strong cue for original images. Excluding this, however, no dominant dependence on the type of image was observed. Average selection rates for non-figurative paintings (abstract) were ranging from 42.0 to 80.0 % with mean of 62.6 %, which are comparable to those for figurative ones ranging from 43.2 to 86.7 % with mean of 68.2 % (t = 0.916, p = 0.372, *Cohen's d* = 0.410).

Next, to clarify the results' dependency on hue, we focused on mean of the hue distributions that are supposed to reflect the typical hues of paintings. We analyzed 80 paintings (20 originals in Set 1 and 2 × four hue-rotations). First, the hue angle of each pixel in an image defined on the a*-b* plane of CIELAB color space was calculated by

$$hue = \tan^{-1}\frac{b*}{a*}, \quad -180 < \ hue\ [\text{degree}] < \ +180$$

and the mean of the hue distribution across the entire image was determined for each painting. **Figure S1** shows the scatter plots of mean of hue distribution (horizontal axis) and average selection rates (vertical axis) for 80 images. First, it is evident that the average selection rates for the originals (blue dots) are located above those for the hue-rotated ones (green, red, and cyan dots), which corresponds to higher selection rates for unrotated original images than rotated ones, as shown in Figs. 4(a) and 5(a). Second, it can be seen that the mean of hue angle is biased towards the 0–90 deg range, regardless of the type of painting or the presence of hue rotation, as previous studies have pointed out (e.g., Tregillus & Webster, 2016; Montagner, 2016). While there was no significant difference in the mean of hue distribution between originals and hue-rotated images (t = 0.293, p = 0.770, *Cohen's d* = 0.076). Therefore, the mean of hue distribution did not explain the superiority of originals observed in the present study.

Tregillus, K. E. M. & Webster, M. A. *Electron Imag* **2016**, 1–8 (2016).
Montagner, C., Linhares, J. M. M., Vilarigues, M. & Nascimento, S. M. C. *J Opt Soc Am* **33**, A170 (2016).

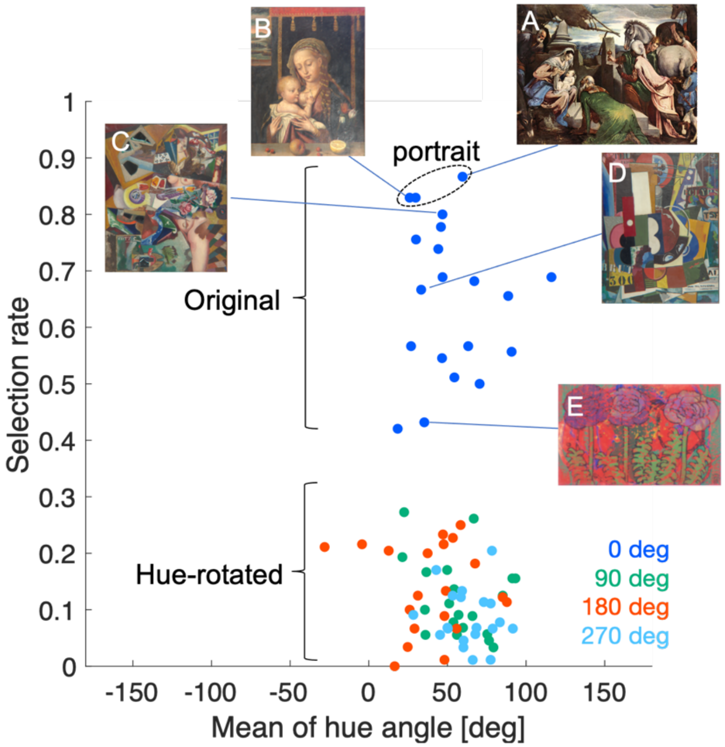

Supplementary Fig. S1. Average selection rates and mean hue angle for images used in the main and replication experiments.

A: oil painting on canvas drawn by *Jacopo Bassano* (ca. 1510 – 1592) in 1569; B: signed by *Wan Kteben* and is from the Renaissance époque painted on wood; C and D: oil paintings on canvas by *Amadeo de Souza-Cardos* (1887 – 1918) in 1917; E: Japanese painting on Japanese paper (*washi*) by *Masayoshi Nakamura* (1924 – 1977) in late 20th century.
